# Characteristics of mutational signatures of unknown etiology

**DOI:** 10.1093/narcan/zcaa026

**Published:** 2020-09-25

**Authors:** Xiaoju Hu, Zhuxuan Xu, Subhajyoti De

**Affiliations:** Rutgers Cancer Institute of New Jersey, New Brunswick, NJ 08901, USA; Rutgers Cancer Institute of New Jersey, New Brunswick, NJ 08901, USA; Rutgers Cancer Institute of New Jersey, New Brunswick, NJ 08901, USA

## Abstract

Although not all somatic mutations are cancer drivers, their mutational signatures, i.e. the patterns of genomic alterations at a genome-wide scale, provide insights into past exposure to mutagens, DNA damage and repair processes. Computational deconvolution of somatic mutation patterns and expert curation pan-cancer studies have identified a number of mutational signatures associated with point mutations, dinucleotide substitutions, insertions and deletions, and rearrangements, and have established etiologies for a subset of these signatures. However, the mechanisms underlying nearly one-third of all mutational signatures are not yet understood. The signatures with established etiology and those with hitherto unknown origin appear to have some differences in strand bias, GC content and nucleotide context diversity. It is possible that some of the hitherto ‘unknown’ signatures predominantly occur outside gene regions. While nucleotide contexts might be adequate to establish etiologies of some mutational signatures, in other cases additional features, such as broader (epi)genomic contexts, including chromatin, replication timing, processivity and local mutational patterns, may help fully understand the underlying DNA damage and repair processes. Nonetheless, remarkable progress in characterization of mutational signatures has provided fundamental insights into the biology of cancer, informed disease etiology and opened up new opportunities for cancer prevention, risk management, and therapeutic decision making.

## INTRODUCTION

**Figure 1.**
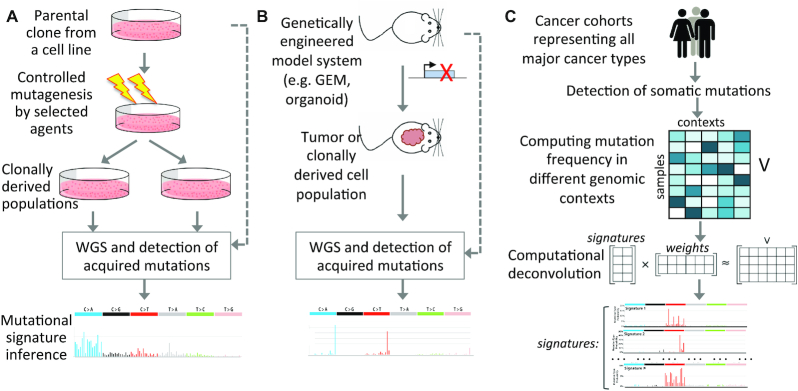
Inferences of mutational signatures using different approaches. (**A**) Targeted mutagenesis using selected agents and sequencing of clonally derived cell populations to identify corresponding mutational signatures. (**B**) Perturbation of selected cellular processes in model systems (e.g. animal models, organoids, cell lines), and then sequencing of tumors or clonally derived cell populations to identify corresponding mutational signatures. (**C**) Data-driven approaches to identify mutational signatures of exo- and endogenous mutagenic processes. While the schematics above are shown for single base substitution (SBS) signatures, similar approaches have also been adopted for doublet base substitution (DBS), small InDel and genomic rearrangement signatures.

Genomic instability is a hallmark of all cancers. Cancer genomes typically harbor 10^3^–10^5^ somatic point mutations, along with other classes of genomic alterations, including insertions and deletions (InDels), copy number variations, rearrangements and ploidy changes (1,2). While a vast majority of somatic mutations are not oncogenic drivers, their patterns of genetic changes and associated contexts can provide insights into past exposure to mutagens, mechanisms of DNA damage and repair defects, and extent of genomic instability, which in turn can guide rational strategies for cancer prevention, risk management and therapeutic decision making (3–5). Targeted mutagenesis and engineered perturbation of cellular processes and reporter assays in cell lines and animal model systems have been widely used to establish the consequences of exogenous mutagenic exposure, as well as endogenous DNA damage and genome maintenance processes [reviewed in (6–9)] (Figure 1A and B). More recently, computational deconvolution of mutational patterns in somatic genomes has provided complementary and unbiased insight into the genome-wide consequences of these mutagenic processes *in vivo* in human tissues. Here, we first describe the computationally derived mutational signatures, emerging bioinformatics resources for analysis of the signatures and characteristics of signatures of known and unknown etiologies; we then discuss the emerging approaches for broader context-guided assessment of somatic mutations, mechanistic inference of the signatures and future direction.

## DATA-DRIVEN INFERENCE OF MUTATIONAL SIGNATURES

Consequences of exposure to carcinogenic agents were known even in ancient civilizations. One of the oldest descriptions of cancer is found in an Egyptian papyrus dated about 3000 BC. After the industrial revolution, coal tar was prescribed for medical purposes in the 1800s, but later it was suspected to cause cancer in animals. In 1915, Yamagiwa Katsusaburo and Koichi Ichikawa experimentally showed that coal tar can induce tumors on rabbits' ears, which could be one of the early systematic experiments demonstrating chemically induced carcinogenesis. However, the idea that carcinogens cause DNA damage did not arise until the 1950s, and the now accepted paradigm of cancer development that cancer is a genetic disease that progresses via mutagenesis began to take shape in the 1960s. Initially, a number of reporter assays were used to investigate mutagenic processes in cell lines and model systems (10–14). However, these were relatively low throughput and did not capture all aspects of the complexity of environmental exposure and deficiency in genome maintenance that are characteristics of the mutational landscapes of human tumors and nonmalignant somatic cells.

In 2010, Pleasance *et al.* used whole genome sequencing to analyze mutational patterns in cancer cell lines (15,16) and reported that lung and skin cancer cell lines show characteristic signatures of smoking and UV exposure, respectively. More recently, whole genome sequencing and whole exome sequencing of thousands of cancer genomes have provided an opportunity to examine mutation patterns in cancer genomes using data-driven approaches and infer their likely etiologies (Figure 1C). Alexandrov *et al.* implemented non-negative matrix factorization (NMF) to deconvolve mutation patterns in cancer genomes and identified an initial set of 30 mutational signatures (17,18). More recently, using multiple computational methods the Pan-Cancer Analysis of Whole Genomes (PCAWG) study has identified a total of 77 consensus mutational signatures, comprising 49 SBS, 11 DBS, 17 InDel and 6 rearrangement signatures (19).

A majority of computationally inferred signatures match with the mutation profile characteristics of known mutagenic processes. These include environmental carcinogens [e.g. smoking, UV, etc. (19)], food-borne mutagens [e.g. aflatoxin (20)] and those attributed to cytotoxic treatments [e.g. cisplatin exposure (21)]. Recently, SBS17 has been attributed to 5-fluorouracil treatment (22). These findings have provided insights into cancer etiology and influenced treatment options. Analysis of mutational patterns has also unveiled novel mutagenic processes and established their etiologies [e.g. kataegis (23) and chromothripsis (24)]. It appears that some mutational signatures typically arise progressively during aging processes in normal somatic cells [e.g. the clock-like signatures (25)], whereas some other signatures (e.g. signatures of burst-like APOBEC mutagenesis and uncorrected replication error) probably arise late during tumor progression (26,27). The PCAWG mutational signature analyses and COSMIC catalog of mutational signatures provide an excellent discourse of the latest mutational signatures, their etiologies and nucleotide-level characteristics (18,19).

## COMPUTATIONAL RESOURCES FOR EXTRACTION AND ANALYSIS OF MUTATIONAL SIGNATURES

A number of computational resources have been developed for extraction, interpretation and annotation of mutational signatures from large-scale somatic mutation data. WTSI (28) and Emu (29) were among the first available to identify mutational signatures from somatic mutation data in cancer genomes. Since then, a number of additional tools such as SomaticSignatures (30), SigProfiler (19), SignatureAnalyzer (19), sigfit (31), Helmsman (32), maftools (33), signeR (34) and others have been developed. These tools use probabilistic approaches and NMF to process and extract mutational signatures *de novo* from cancer genomic data. A subset of these can now identify signatures associated with other classes of genomic alterations such as InDels, DBS and/or rearrangements. The number of mutational signatures present in somatic genomes is not known *a priori*; some tools can automatically estimate an optimal number of signatures [e.g. EMu (29), maftools (33)]. Appropriate null models are critical for meaningful discovery of mutational signatures from genomic data. Bergstrom *et al.* (35) have developed utilities to simulate mutational landscapes under different null models, which can be used to examine whether mutational patterns observed in somatic cells show significant enrichment of certain signatures compared to that expected by chance. Together, these tools provide a rich resource for signature discovery. Omichessan *et al.* have tabulated several software and compared their performance for *de novo* signature extraction using simulated and real data, and found that identification of signatures is challenging in tumor genomes comprised of multiple signatures each having modest contributions and that probabilistic approaches tend to perform better than other approaches (36). In the future, it might be appropriate to have a DREAM Challenge-type community-driven systematic study to compare and benchmark performance of these tools on an open platform.

A number of computational methods such as deconstructSigs (37), sigfit (31), MutationalPatterns (38), decompTumor2Sig (39), etc. allow users to determine relative contributions of the signatures from an existing catalog in the set of somatic mutations in tumor genomes. Some of them [e.g. SignatureEstimation (40), SigsPack (41)] further allow estimation of confidence intervals for each identified signature in a somatic genome. Some utilities such as MuSiCa (42) and MutaGene (43) allow web-based analysis of mutational signatures.

It appears that analyzing somatic mutations in their genomic context and local patterns can provide additional critical insights. Singh *et al.* used an hidden Markov model (HMM)-based approach to identify different mutagenesis-related composite epigenomic contexts, and then used that to identify patterns of mutation signatures in different contexts and conclude that SBS8 likely arises due to uncorrected late replication errors (44). TensorSignatures (45) has been recently developed based on an overdispersed statistical model incorporating mutational catalogues, transcription and replication strand bias, and kataegis, leading to more robust extraction of mutation signatures. SigMa (46) and recently StickySig (47) model statistical dependencies among neighboring mutations to characterize strand coordination, and other genomic and nongenomic factors that influence the activity of mutation signatures. Such efforts are exciting and contributing to the broader understanding of the patterns of the mutational signatures in the genome. For example, it appears that some signatures (e.g. APOBEC mutagenesis) are associated with extended processivity (48).

Other resources have been developed to link the mutational signatures with tumor evolution and therapeutic strategies. Temko *et al.* used a probabilistic approach to identify the preference for oncogenic mutations given the prevailing mutational signatures (49). MutaGene offers a maximum likelihood approach to predict the likely etiology of individual mutations, which can be used to infer the likely mutagenic process behind individual driver mutations in a cancer (50). Palimpsest (51) and trackSig (52) can provide clonality inferences for mutational signatures, which can inform how mutagenic processes change during the course of tumor progression. Structural variation signatures (53) and HRDetect (54) can identify homologous recombination (HR) deficiency in human tumors, which could be targeted clinically. Several other tools can predict signatures (e.g. APOBEC signature) associated with cancer diagnosis and/or guide suitable treatment, including immunotherapy (7,55,56).

## MUTATIONAL SIGNATURES OF KNOWN AND UNKNOWN ETIOLOGIES

Etiologies of nearly one-third of the COSMIC version 3 signatures are not yet fully understood as of July 2020. Some signatures (e.g. SBS3, SBS8, SBS5) correlate with important clinical and molecular features, but their underlying mechanisms are not yet fully determined or remain debated (18). Are the known and unknown signatures somewhat different? Are there certain characteristics or lack thereof that helped decipher the known signatures, and could those provide potential informed guidance while investigating the signatures of hitherto unknown etiologies?

The COSMIC mutational signatures were identified using NMF, which is a mathematical technique for blind source separation, resolving an original matrix into a product of two matrices with lower dimensions (57). It has an inherent clustering property such that it implicitly and parsimoniously groups the original dataset into a smaller set of relatively homogeneous subgroups. Thus, if mutations of a given etiology are sparse and clustered, i.e. occur in selective nucleotide contexts, affect a select subgroup of patients and have high attributed mutation burden, it would be easier to identify its associated signature by NMF-based deconvolution. Indeed, many well-established endo- and exogenous mutational signatures have these properties. Moreover, many of these signatures are associated with external mutagenic exposure or oncogenic mutations in DNA repair pathways that result in a specific and substantial burden of associated mutations in tumor genomes, which are associated with clinical variables. For instance, smoking and UV exposure cause an excessive burden of somatic mutations with distinct substitution patterns in lung and skin cancer subtypes. Some DNA repair and genome maintenance defects also result in distinct nucleotide-level changes and manifest in tissue-dependent manner. For instance, tumors with mismatch repair defects (e.g. MSH2, MLH1 and MSH6 mutations) or DNA polymerase functions (e.g. POLD1 and POLE mutations) are relatively common in colon tumors, which lead to distinct substitution biases and up to one to two orders of magnitude more mutations in affected tumors compared to other tumors of the same subtypes; notably, a minority of tumors possessing defects in both mismatch repair and DNA polymerase functions show intricate signatures defined by SBS14 and SBS20 (58,59). The other major subset of interpretable mutational signatures of endogenous origin often involves sporadic, burst-like activity of specific mutations at distinct contexts (e.g. APOBEC signatures) (19), such that the affected genomes have substantial burden of associated signatures (26,48). Indeed, these signatures were among the first to be identified. Rigorous examination, curation and validation by the broader collaborative scientific community in general, and the COSMIC initiative in particular, have helped establish etiologies of more complex signatures (18). The success of this approach is exemplified by deciphering complex signatures (e.g. SBS3, SBS5, SBS25 or SBS35) that have nonspecific tissue and/or nucleotide context preferences.

**Figure 2.**
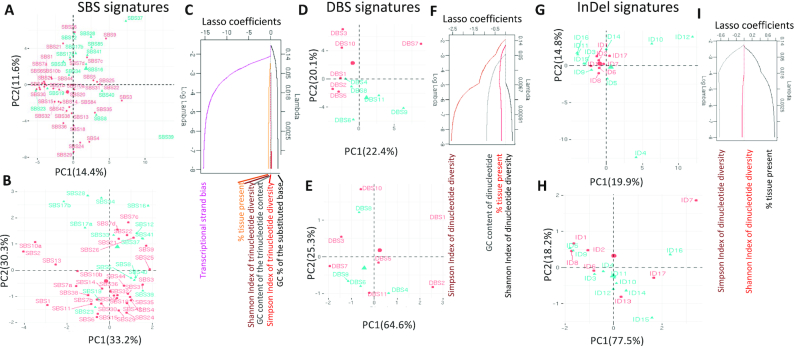
Differences between known and hitherto unknown SBS, DBS and InDel mutational signatures. PCA plots showing known (pink) and unknown (cyan) SBS signatures based on their (**A**) trinucleotide frequencies, and (**B**) multiple features including GC content, Shannon’s and Simpson’s diversity indices of trinucleotide context usage, transcriptional strand bias and presence in proportions of cancer types that could be computed based on their COSMIC signature information alone. (**C**) Coefficients of the features with decreasing lambda in a LASSO regression are shown. PCA plots showing known (pink) and unknown (cyan) DBS signatures based on their (**D**) dinucleotide frequencies, and (**E**) multiple features including GC content, Shannon’s and Simpson’s diversity indices of dinucleotide context usage and presence in proportions of cancer types. (**F**) Coefficients of the features with decreasing lambda in a LASSO regression are shown. PCA plots showing known (pink) and unknown (cyan) InDel signatures based on their (**G**) nucleotide frequencies, and (**H**) multiple features including Shannon’s and Simpson’s indices and presence in proportions of cancer types. (**I**) Coefficients of the features with decreasing lambda in a LASSO regression are shown. In all cases, random forest mean decrease in Gini index and mean decrease in accuracy, which indicate feature importance, also showed comparable patterns.

We further examined whether there are other quantitative differences between the known and hitherto unknown signatures. When the SBS signatures were projected on a principal component analysis (PCA) plot based on their trinucleotide frequencies (Figure 2A), the signatures of unknown etiologies partially segregated from the known signatures, although SBS7c, SBS7d and SBS22 showed contextual similarities. In contrast, signatures SBS8, SBS40, SBS19, etc. were more similar to known signatures. Next, for each signature we analyzed a number of meta-features including GC content, transcriptional strand bias, presence in proportions of cancer types and diversity of trinucleotide preference using Shannon’s and Simpson’s indices (Figure 2B; also see the Supplementary Data for details of analyses) that could be computed directly from the COSMIC signatures without additional data on broader (epi)genomic or tissue contexts. When the signatures were projected on a PCA plot using these meta-features, the unknown signatures partially segregated from the known signatures and the overall differences were qualitatively similar to those observed above. Such differences are not due to technical issues in NMF-based signature extraction; the SBS signatures are generally robust between COSMIC versions and have been identified by multiple algorithms (19). Feature selection using LASSO indicated that transcriptional strand bias and GC content are associated with differences between signatures of known and unknown etiologies (Figure 2C); the unknown signatures on average have weak transcriptional strand bias and lower GC content. When the analyses were extended for the DBS and InDel signatures (Figure 2D–I), the signatures of known and unknown etiologies showed some differences in their nucleotide context usage (Figure 2D and G), while at the level of meta-features the differences were less apparent (Figure 2E and H). The known DBS signatures were characterized by CC>NN (DBS1 and DBS2), TT>NN (DBS3 and DBS7) and CG>NN (DBS10), while unknown were dominated by GC>NN, TG>NN and AC>NN substitutions. Nucleotide diversity (Simpson’s index) at mutated positions and GC content were important for discriminating DBS signatures of known and unknown etiologies, while nucleotide diversity (Simpson’s index) at mutated positions was relevant for a similar analysis on the InDel signatures. Both DBS and InDel signatures are recent, such that differences between the known and hitherto unknown signatures may be superficial, and etiologies of many of them could be established in the near future.

It is possible that the unknown mutational signatures, especially those with rare occurrence and/or modest effect sizes, might need larger sample sizes for robust detection. However, compositions of the major signatures are broadly consistent across COSMIC versions (18), indicating that these are usually distinct and stable (19). It is also possible that signatures of basal genome maintenance, DNA damage and repair processes that are operative in most somatic cells during development and aging are inter-related (59), and thus harder to isolate. Differences in transcriptional strand bias and/or GC content (Figure 2) raise a provocative question whether many, though not all, hitherto unknown signatures might predominantly occur outside gene regions, which are relatively poorly characterized. Moreover, crosstalk between multiple genome maintenance processes might lead to complex signatures. For instance, co-occurrence of mismatch repair (MMR) defect and DNA polymerase mutations results in a signature (SBS14 and SBS20) that is distinct from both (58,59). It is also possible that mutations arising from dose-dependent or reduced activities of genome maintenance processes might be harder to pinpoint than those associated with oncogenic mutations in DNA replication and repair-related genes. Multidisciplinary efforts from the scientific community are addressing these open questions from different angles, and their innovative approaches are expected to provide new insights into origins, higher order patterns and consequences of the mutational signatures, as we discuss in the following sections.

## BROADER CONTEXT-GUIDED ASSESSMENT OF SOMATIC MUTATIONS

**Figure 3.**
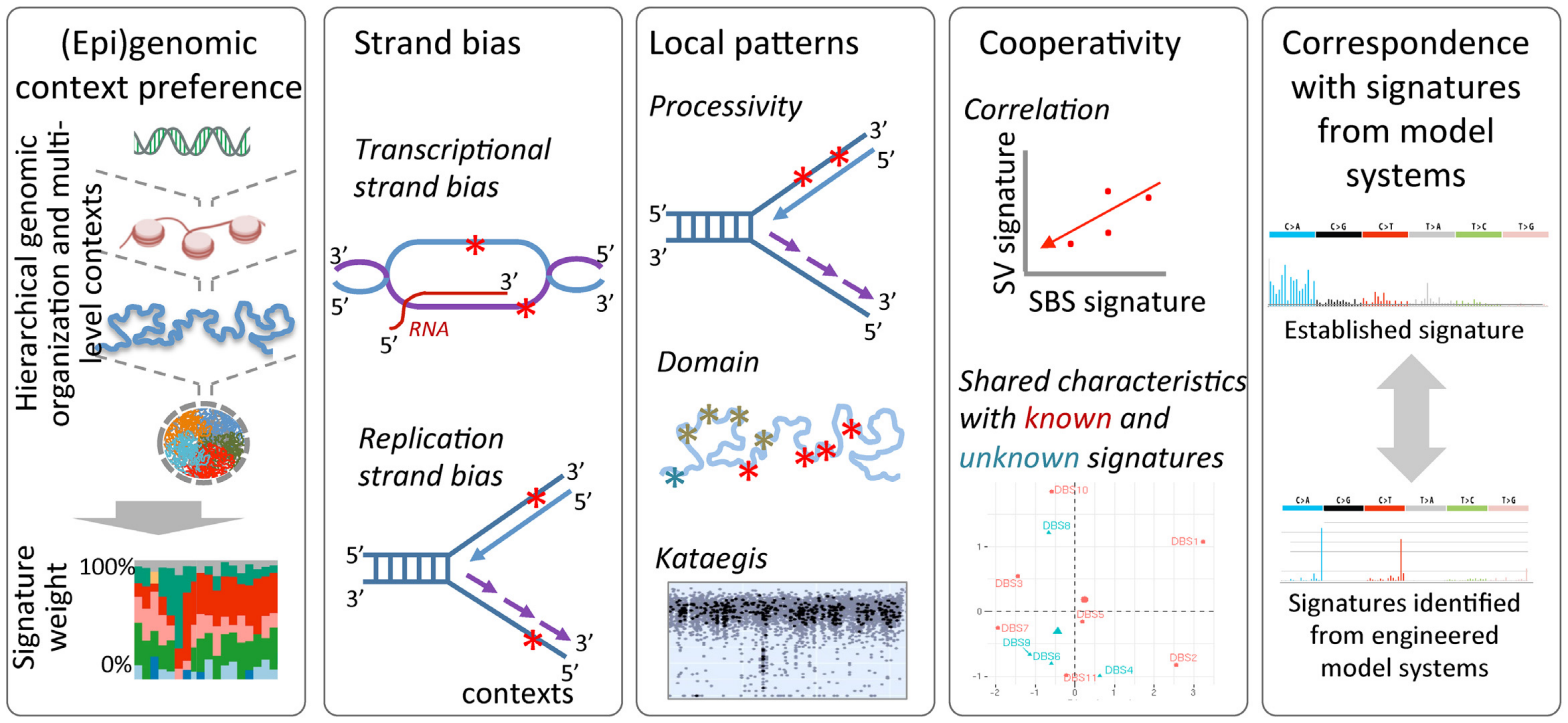
Emerging strategies for investigating characteristics of mutation signatures. Approaches such as analyses of strand bias, context preference, local patterns of mutations, cooperativity and correspondence with laboratory-generated mutation signature can potentially help provide additional mechanistic insights into the mutational signatures, including those of hitherto unclear origin. Only representative examples are shown.

DNA damage and repair depend on local nucleotide sequences, as well as broader genomic, epigenomic and nuclear contexts. Mechanisms underlying some mutational signatures might be sufficiently explained by their nucleotide contexts alone [e.g. tobacco signature (60)], while in other cases broader genomic and epigenomic contexts, which include chromatin, replication timing, processivity and other relevant features, may help understand the mechanisms of DNA damage and DNA repair processes (Figure 3). COSMIC version 3 signatures already consider transcriptional and replication strand biases (18). Transcriptional strand biases can inform whether transcription-coupled DNA damage and repair processes could contribute to the signature of interest. Likewise, replication strand bias can help predict whether replication of continuous strands and Okazaki fragments or other associated factors could potentially contribute to a signature of interest.

Chromatin and nuclear contexts can influence mutagenesis and DNA repair pathway choices (61–63), such that certain signatures may show context-specific enrichment. To narrow down likely mechanisms for a signature of interest, under-representation of the signature in certain contexts and absence of corresponding context-associated biases can help exclude unlikely possibilities. For instance, using chromatin and replication timing data it was shown that SBS8 is uncommon in gene-rich euchromatin regions, and likely arises in late and fast replicating regions due to uncorrected replication errors during tumor progression (44). Local associative patterns of mutations can suggest potential cooperative processes driving the mutation signature(s). A classic example is APOBEC mutagenesis: it was shown that signatures SBS2 and SBS13 occur in late and early replicating regions, respectively, and show significantly long stretches of processivity that might be due to sporadic but burst-like APOBEC mutagenesis during replication stress (48).

Signatures attributed to the same underlying mutagenic processes may correlate within and between individuals. For instance, SBS signature SBS3, InDel signatures ID6 and ID8, and rearrangement signatures SV3 and SV5 indicate different aspects of defects in homologous recombination-mediated repair (54). Similarly, SBS8 and SBS40 show comparable trinucleotide frequencies and similar context preferences, and may be related (44). It is possible that attributes of other unknown signatures could be predicted from their association with known signatures. Moreover, correspondence analysis between mutational signatures generated in engineered model systems with those in human tumors can help establish etiologies of specific signatures (64,65), as also discussed below. In light of these observations, it is unlikely that a single strategy will be necessary and sufficient to explain all signatures, and those signatures that are not sufficiently explainable by trinucleotide contexts alone could benefit from analyses of broader contexts and patterns.

## MECHANISTIC INFERENCE OF MUTATIONAL SIGNATURES

Previous works on mutational landscape of tumor genomes and mutational signatures have primarily analyzed epigenomic contexts from closely related cell types or those contexts that are cell type invariant (8,48,63,66,67). Unfortunately, data on epigenome and replication profile are limited to reference cell lines and tissues (68,69), and it remains technically challenging to obtain similar high-quality data from primary cell types, especially from rare cell populations from normal or tumor tissues, which may have genetic and nongenetic heterogeneity. Emerging single-cell assays are enabling multi-omics profiling on primary cell populations (70,71), allowing for integrating relevant epigenomic and mutation data directly from the cell populations of interest, which may provide valuable insights about mutagenic processes in somatic cells *in vivo*.

Reporter assays in well-characterized cell lines and model organisms can validate selected mutational signatures and provide mechanistic insights. After the development of massively parallel sequencing technologies, targeted or whole genome sequencing of clonally derived cell populations has been used to complement reporter assays and determine genome-wide consequences of the mutagenic processes at base-pair level resolution. It enables detailed characterization of mutational signatures of the targeted mutagenic process and allows one to directly connect the findings with clinical observations in cancer and other diseases. Using this approach, Szikriszt *et al.* analyzed the effects of eight common cytotoxic agents, including cisplatin, to show that select agents can cause significant mutagenesis with distinct mutational signatures (72). More recently, a larger compendium study analyzed the effects of 79 known or suspected environmental carcinogens by analyzing mutational patterns in isogenic cell populations with controlled exposure (65). In parallel, other studies have focused on analyzing mutational signatures of defects in DNA replication, DNA damage response or DNA repair pathways. Póti *et al.* used the CRISPR–Cas9 system to disrupt key homologous recombination-mediated repair and checkpoint genes, and also correlated their genomic mutagenic phenotypes with drug sensitivity (64).

Cell line-based systems are easy to manipulate and can recapitulate clinically relevant mutation signatures (9), but design of experiments requires careful considerations. Inherent chromosomal and genomic instability in some common cell lines can potentially result in genetic heterogeneity within the initial cell population and/or accumulation of spontaneous mutations during passage, such that without appropriate control these mutations can introduce biases in the predicted mutational signature of the targeted mutagenic process. Therefore, it is important to select cell lines with relatively higher level of genomic stability, and to sequence clonally amplified, isogenic cell populations after exposure. Stable cell lines such as DT40 and RPE1 remain popular choices. Furthermore, DNA damage response and DNA repair defects can have cell type-dependent consequences in certain contexts. For instance, germline mutations in homologous recombination-mediated repair (e.g. BRCA1, BRCA2), mismatch repair (e.g. MLH1, MSH2, MSH6) or genome maintenance (e.g. TP53) result in increased cancer incidence rate in selected tissue types. Therefore, it is necessary to use relevant cell lines to examine physiologically relevant consequences. Kucab *et al.* used a human induced pluripotent stem cell line, which is noncancerous, undifferentiated and diploid, and has stable karyotype (65). Development of other cell lines with similar robust characteristics will be of interest to the scientific community.

Animal models are key components of mechanistic studies. Generation of novel animal models requires substantial efforts, and although key cellular processes are largely conserved among higher eukaryotes (e.g. mammals), it still remains nontrivial to sufficiently recapitulate the consequences of multifaceted exposure, human physiology and aging in the model systems in laboratory environments. Nonetheless, recent developments are exciting. Recently, Jacks and colleagues have adapted the CRISPR–Cas9 system in a mouse model of small cell lung cancer to rapidly model mutations in target genes (73), and such models can be potentially used to examine the genome-wide consequences of loss of key DNA repair genes *in vivo*. Application of optogenetics has enabled detection of chromosome dynamics in response to accumulating DNA damage in zebrafish (74). In any case, the laboratory-based model systems will continue to provide fundamental mechanistic insights into DNA replication and repair defects in cell and tissue contexts and put them in the perspective of development, aging and diseases such as cancer.

## FUTURE DIRECTIONS

Pan-cancer studies have identified the stable and reproducible catalog of mutational signatures for all common types of genomic alterations, from tumor samples representing all major cancer types, and also established the etiologies of a subset of the signatures (19). Degasperi *et al.* provided a practical framework outlining necessary precautions and rational approaches for careful analyses of mutational signatures (8). This probably marks the end of the initial discovery phase of the prevalent mutation signatures in somatic tissues, and over the next few years we anticipate further refinement and consolidation of the signatures, and the focus will turn to gain deeper mechanistic insights into their etiologies using interdisciplinary approaches. Cell line and other laboratory model systems that take advantage of emerging technologies will advance the validation and mechanism-oriented investigations. Advancement of single-cell genomics technologies will probably enable more robust detection of somatic mutations in single cells or small number of subclonal cell populations in malignant or normal tissues (75). Long InDels and complex structural variations are still not well characterized. Long and linked read technologies will probably help reconstruct complex genomic alterations better (76) and provide testable hypotheses about their etiologies.

Early tumor development remains asymptomatic, such that early stages of genomic instability and neoplastic changes *in vivo* in humans are challenging to study (77). Analysis of accumulation of mutational signatures during development, premalignant and malignant contexts can provide some insights into cancer initiation and progression. Predominant mutational processes in tumor genomes can influence selection of oncogenic driver mutations (49), ultimately guiding the course of cancer development. Such a bias might also be relevant for emergence of resistant mutations during treatment. On the other hand, mutational signatures provide information about genome maintenance defects in individual tumors that can be exploited in precision medicine settings. For instance, deficiency in homologous recombination-mediated repair tends to sensitize tumors to PARP inhibitors, while tumors with excessive somatic mutation burden due to APOBEC activity and defects in mismatch repair and DNA polymerase functions tend to be responsive to immunotherapy (7,54,55). Current progress in these areas is encouraging (3–5), and may provide additional options for combination therapies targeting multiple types of cancer. Taken together, mutational signatures derived from somatic mutations, a majority of which are passenger mutations, have provided fundamental insights into the biology of cancer and disease etiology, and have opened up opportunities for clinical intervention that are truly remarkable.

## Supplementary Material

zcaa026_Supplemental_File
