# Supplementary material for: Characteristics of mutational signatures of unknown etiology
Source: NAR Cancer. 2020 Sep 25;2(3):zcaa026. doi: 10.1093/narcan/zcaa026 (PMC7520824; doi:10.1093/narcan/zcaa026)
Supplement: zcaa026_Supplemental_File [file zcaa026_supplemental_file.pdf]

Supplementary Data
for
Characteristics of mutational signatures of unknown etiology

Xiaoju Hu, Kellen Xu, Subhajyoti De

Supplementary Methods

The catalog of mutational signatures (SBS, DBS and ID, COSMIC v3.1) was obtained from Alexandrov et al. Nature 2020(1), which used sigProfiler to analyze somatic mutations from whole genome sequencing of tumors from multiple major cancer types included in the PCAWG Consortium to derive the signatures. Patterns of transcriptional strand bias for these signatures are available at the Synapse (https://www.synapse.org/#!Synapse:syn12009743). The list of the tumor tissue-types the signatures are present are listed at https://www.synapse.org/#!Synapse:syn11804040. Possible sequencing artefacts signatures were removed. All features were measured based on 96 tri-nucleotide contexts of each mutational signature, except for percent tissue prevalence, which was derived from the summary of the COSMIC v3.1 signatures present in the tumor types in the PCAWG Consortium.

The diversity of trinucleotide context usage for the mutational signatures was measured using Shannon's and Simpson's indices as implemented in the vegan(2) R package. Transcriptional strand bias was calculated as $\sum_{k=1}^{96} (T-U)^2/96$. T: Transcription strand, U: Untranscription strand. For each SBS signature, overall GC content of the trinucleotide context was estimated as: $\sum_{k=1}^{96} GC_k \times f_k$ where GC_k is the GC content of each tri-nucleotide context and f_k is the percentage of single base substitutions at that context in that signature. GC content of the substituted base was estimated in a similar manner after only considering GC content of the middle base that underwent single base substitution. Principal component analysis (PCA) was performed using mutation catalog frequencies and all aforementioned features through the functions of prcomp and fviz_pca_ind implemented in factoextra package (3).

We classified the SBS signatures into two categories - 'known' and 'unknown' as reported in the COSMIC SBSv3.1, and used Random Forest and Lasso to identify the features that might be most informative to discriminate the two groups. Mean decrease accuracy and mean decrease Gini index were calculated using randomForest package (4), and the magnitude of coefficient for each feature was measured through glmnet package (5). We extended the analyses to the DBS and InDel signatures, classifying them as 'known' and 'unknown' as reported in the COSMIC v3.1, and then used Random Forest and Lasso to identify the context features that might be informative to discriminate the two groups. Data on some features was relevant for only specific classes of signatures (e.g. length of InDels).

References

1. Alexandrov, L.B., Kim, J., Haradhvala, N.J., Huang, M.N., Ng, A.W.T., Wu, Y., Boot, A., Covington, K.R., Gordenin, D.A., Bergstrom, E.N. *et at.*(2020). The repertoire of mutational signatures in human cancer. *Nature*, ***578***, 94-101.
2. Dixon, P. (2003). VEGAN, a package of R functions for community ecology. *J. Veg. Sci.*, **14**, 927-930.
3. Kassambara, A., & Mundt, F. (2017). Package 'factoextra'. Extract and visualize the results of multivariate data analyses, 76.
4. Breiman, L. (2015). randomForest: Breiman and Cutler's random forests for classification and regression. R package version, 4, 6-12.
5. Friedman, J., Hastie, T., & Tibshirani, R. (2010). Regularization paths for generalized linear models via coordinate descent. *J. Stat. Softw.*, **33**, 1.

## determine shannon and simpson diversity for SBS, and the same code available for DBS and ID when replacing input file as “sigProfiler_DBS_signatures.csv” or “sigProfiler_ID_signatures.csv”

```
library(vegan)
x<-read.table("~/Documents/sigProfiler_SBS_signatures.csv",header=T,sep=",",check.names=F)
x<-x[,c(-1,-34,-50,-52:-69)] # remove possible sequencing artefacts signatures for SBS
shannon<-c()
for (i in 3:ncol(x)){a<-diversity(x[,i], index = "shannon");shannon<-rbind(shannon,a)}
simpson<-c()
for (i in 3:ncol(x)){a<-diversity(x[,i], index = "simpson");simpson<-rbind(simpson,a)}
```

## determine transcription bias for SBS

```
x<-read.table("~/Documents/sigProfiler_TSB_signatures.csv",header=T,sep=",",check.names=F)
x<-x[,c(-35,-51,-53:-68)] # remove possible sequencing artefacts signatures for SBS
trans_bias<-c()
for (i in 1:96){y<-x[i,]-x[(i+96),];z<-y*y;trans_bias<-rbind(trans_bias,z)}
trans_bias<-as.data.frame(colSums(trans_bias)/96)
```

## determine mono-GC content for SBS

```
x<-read.table("~/Documents/sigProfiler_SBS_signatures.csv",header=T,sep=",",check.names=F)
x<-x[,c(-1,-34,-50,-52:-69)] # remove possible sequencing artefacts signatures for SBS
GC_mono<-c()
for (i in 3:ncol(x)){a<-test[c(1:48),i]*0.33;GC_mono<-cbind(GC_mono,a)}
GC_mono<-as.data.frame(colSums(GC_mono))
```

## determine tri-GC context for SBS, the same code available for DBS when replacing input file as “sigProfiler_DBS_signatures.csv”

```
x<-read.table("~/Documents/sigProfiler_SBS_signatures.csv",header=T,sep=",")
x<-x[,c(-1,-34,-50,-52:-69)] # remove possible sequencing artefacts signatures for SBS
count.string<-function(strings, pattern){
                    counts<-NULL
                              for(i in 1:length(strings)){
                                        counts[i]<-length(attr(gregexpr(pattern,strings[i])[[1]],
                                                  "match.length")[attr(gregexpr(pattern,strings[i])[[1]], "match.length")>0]) }
                                        return(counts)}
c<-c()
library(stringr)
for (i in 3:ncol(x)){
          a<-str_split_fixed(x$SubType,"",3)[,2];b<-
          (count.string(strings=a,pattern="C")+count.string(strings=a,pattern="G"))/2;y<-x[,i]*b;c<-cbind(c,y)}
GC_tri<-as.data.frame(colSums(c))
```

## determine tissue percentage for SBS, the same code available for DBS and ID when replacing input file as “PCAWG_sigProfiler_DBS_signatures_in_samples.csv” or “PCAWG_sigProfiler_ID_signatures_in_samples.csv”

```
x<-read.table("~/Documents/PCAWG_sigProfiler_SBS_signatures_in_samples.csv",header=T,sep=",”)
x<-x[,c(-35,-51,-53:-68)] # remove possible sequencing artefacts signatures for SBS
tissue_ratio<-c()
for(i in 4:ncol(x)){
a<-length(unique(x[which(x[,i]!=0),1]))/length(unique(x$Cancer.Types));tissue_ratio<-rbind(tissue_ratio,a)}
```

## combined all available features into one file named "SBS_features" with one column annotation of known and unknown etiology mutation signatures. The same step was done by DBS and ID.

## PCA plot using mutation catalog frequencies for SBS, the same code available for DBS and ID when replacing input file as “sigProfiler_DBS_signatures.csv” or “sigProfiler_ID_signatures.csv”

```
SBS<-read.table("~/Documents/sigProfiler_SBS_signatures.csv",header=T,sep="\t",stringsAsFactors=FALSE)
SBS<-SBS[,c(-1,-34,-50,-52:-69)] # remove possible sequencing artefacts signatures for SBS
SBS96 <- data.frame(t(SBS[-1]))
colnames(SBS96) <- SBS[, 1]
SBS96_rm<-SBS96[,-97]
SBS96_rm[] <- lapply(SBS96_rm, function(x) as.numeric(as.character(x)))
data<- as.matrix(SBS96_rm)
res.pca <- prcomp(data, scale = TRUE)
fviz_pca_ind(res.pca, habillage=SBS$type,textsize = 1,repel = TRUE)
```

## PCA plot using all available features for SBS, the same code available for DBS and ID when replacing input files as "SBS_features", or "ID_features".

```
x<-read.table("~/Documents/SBS_features",header=T,sep="\t",stringsAsFactors=FALSE,row.names=1)
x$type<-ifelse(x$type==1,"Known","Unknown")
x<-x[,-1]
x_rm[] <- lapply(x_rm, function(x) as.numeric(as.character(x)))
library(factoextra)
data<- as.matrix(x_rm)
res.pca <- prcomp(data, scale = TRUE)
fviz_pca_ind(res.pca, habillage=x$type,textsize = 1,repel = TRUE)
```

## random forest to determine mean decrease accuracy and mean decrease Gini for SBS, the same code available for DBS and ID when replacing input file as "SBS_features", or "ID_features".

```
library(randomForest)
test<-read.table("~/Documents/SBS_features",header=T,sep="\t")
test<-test[,-]
test$type<-as.factor(test$type)
rf <- randomForest(type ~.,data=test)
model.rf <- randomForest(type~., test, importance=TRUE)
varImpPlot(model.rf,main="SBS Mutation signature random forest")
```

## Lasso regression to determine the magnitude of coefficient for each feature for SBS, the same code available for DBS and ID when replacing input files as "SBS_features", or "ID_features".

```
x<-read.table("~/Documents/SBS_features",header=T,sep="\t",stringsAsFactors=FALSE,row.names=1)
library(glmnet)
mod <- glmnet(as.matrix(x[c(-1,-2)]), x[,2])
plot_glmnet(mod)
```
